# Prevalence of Vitamin D Deficiency Among Infants in Northern India: A Hospital Based Prospective Study

**DOI:** 10.7759/cureus.11353

**Published:** 2020-11-05

**Authors:** Swathi Chacham, Swati Rajput, Shilpa Gurnurkar, Anissa Mirza, Vartika Saxena, Senkadhirdasan Dakshinamurthy, Jaya Chaturvedi, Jagdish P Goyal, Madhuradhar Chegondi

**Affiliations:** 1 Pediatrics, All India Institute of Medical Sciences, Rishikesh, IND; 2 Biochemistry, All India Institute of Medical Sciences, Rishikesh, IND; 3 Pediatric Endocrinology, Nemours Children's Hospital, Orlando, USA; 4 Community and Family Medicine, All India Institute of Medical Sciences, Rishikesh, IND; 5 Obstetrics and Gynaecology, All India Institute of Medical Sciences, Rishikesh, IND; 6 Pediatrics, All India Institute of Medical Sciences, Jodhpur, IND; 7 Pediatrics, University of Iowa Stead Family Children's Hospital, Iowa City, USA

**Keywords:** infants, vitamin-d deficiency, hypocalcemia, neonates, maternal

## Abstract

Background: Vitamin D deficiency is one of the major nutritional deficiencies and an important contributor to nutritional and growth failure in infants, especially in those with low socioeconomic status.

Aim: The primary objective of this study was to determine the proportion of vitamin D deficiency in infants, and the secondary objective was to assess the correlation between infant and maternal vitamin D levels.

Methods: This prospective, observational study was carried out at a tertiary care center, All India Institute of Medical Sciences in Rishikesh, Uttarakhand, India, in the Department of Pediatrics from January 2017 to December 2018. Children aged less than one year and their mothers were enrolled in the study. All the infants attending the Department of Pediatrics for well-child visits and sick-child visits were enrolled after obtaining written, informed consent. Infants with major congenital malformations and liver and kidney dysfunction were excluded. Serum vitamin D level of <20 ng/mL was defined as vitamin D deficiency.

Results: A total of 200 infants and 200 mothers were enrolled in the study. Among the study infants, 80% were neonates, and 20% were infants beyond the neonatal period. The prevalence of vitamin D deficiency was 74% in infants and 85.5% in mothers. Nearly half of the infants and mothers had severe vitamin D deficiency. Logistic regression analysis showed a positive correlation between maternal and infant vitamin D levels (r=0.074, p<0.001) and also with neonatal age group and low socioeconomic status. Hyperphosphatemia and hypocalcemia were predominant biochemical manifestations.

Conclusion: The prevalence of vitamin D deficiency among the study infants was 74%. Neonatal age group, lower socioeconomic status, and maternal vitamin D deficiency were major determinants of vitamin D deficiency in infants.

## Introduction

Vitamin D deficiency is one of the important nutritional deficiencies in children worldwide [1]. It was reported to occur among 96% of infants and 84% of pregnant women by the International Osteoporosis Foundation (IOF) in 2009 [2]. Vitamin D plays a key role in calcium absorption, bone mineralization, and aids in phosphate and magnesium metabolism [1]. The US Endocrine Society defined vitamin D deficiency as serum 25(OH) vitamin D levels less than 20 ng/mL and vitamin D insufficiency as serum 25(OH) vitamin D levels between 21 to 29.9 ng/mL [3]. Globally, it is estimated that around one billion people belonging to all age groups have vitamin D deficiency or insufficiency. In India, 50-90% of children suffer from vitamin D deficiency or insufficiency predominantly due to lower dietary intake of calcium coupled with dark skin color and lack of adequate exposure to sunlight [4,5].

Exclusive breastfeeding in the setting of maternal vitamin D deficiency, reduced dietary consumption, and decreased exposure to sunlight due to seasonal variation are leading causes of vitamin D deficiency among infants [4]. In severe cases, it manifests as rickets, characterized by frontal bossing, widening of wrists, bowing of legs, spontaneous fractures in infants, and can also lead to bony deformities, short stature, muscle weakness, and muscle pain in older children. Apart from skeletal manifestations, vitamin D deficiency can also present with growth failure, recurrent respiratory infections, and developmental delay [4]. Infants with malabsorption syndrome, cystic fibrosis, and nephrotic syndrome are at increased risk [4].

The key biochemical changes in infants with vitamin D deficiency consist of hypocalcemia, hyperphosphatemia, and secondary hyperparathyroidism [4,5]. Recommended treatment for neonates with vitamin D deficiency includes 400-1000 international units (IU) of daily vitamin D for 8-12 weeks. Likewise, infants beyond the neonatal period require 1000-5000 IU/day of vitamin D for 8-12 weeks. Timely detection and treatment of vitamin D deficiency are crucial to prevent bony deformities and curtail respiratory morbidity [5]. The paucity of data on vitamin D deficiency status in neonates and infants from Uttarakhand, a northern state of India, prompted the present study.

## Materials and methods

This prospective, observational study was carried out at a tertiary care center, All India Institute of Medical Sciences in Rishikesh, Uttarakhand, India, in the Department of Pediatrics from 2017 to 2018. All the consecutive infants (aged ≤1 year) who came to pediatric outpatient and inpatient units for sick- or well-child visits were enrolled after obtaining written, informed consent. Infants with major congenital malformations, liver and kidney dysfunction, malabsorption syndrome, and those on tube feeding were excluded. Serum vitamin D level of <20 ng/mL was defined as vitamin D deficiency [2]. All the study infants were clinically evaluated by using a predesigned, pretested proforma. Each infant’s anthropometry, general physical examination findings, alongside clinical signs of hypocalcemia (jitteriness, tetany, etc.) and vitamin D deficiency (rachitic rosary, craniotabes, etc.), were monitored and recorded. Maternal details comprising socioeconomic status were recorded. The modified Kuppuswamy scale was used to classify the socioeconomic status [6]. From the enrolled infants, 2 ml of venous blood was collected and analyzed for calcium, phosphorus, alkaline phosphatase, and 25-(OH) vitamin D levels. In addition, serum 25-(OH) vitamin D levels were also estimated in all the mothers of the enrolled infants. Vitamin D levels were analyzed by the chemiluminescence method using Siemens Advia Centaur XP (Siemens Diagnostics, Tarrytown, NY, USA). The research study was approved by the institution’s review board (IM/RC104/2016/29), and written informed consent was obtained from the parents before study enrollment.

Sample size calculation

Considering a 60% prevalence of vitamin D deficiency, 80% power, and an allowable error of seven, we required 196 infant-mother pairs. We enrolled 200 infant-mother pairs for the study.

Statistical analysis

Descriptive statistics were used for calculating percentages, proportion, mean (standard deviation (SD)), and median (inter-quartile range (IQR)). Means of continuous variables between groups were compared by Student's t-test. Correlation between variables was assessed by using Pearson’s correlation coefficient. Binary logistic regression analysis was performed simultaneously for finding the determinants of vitamin D deficiency. A significance level of 5% was used for all of the statistical tests. The data was analyzed using Statistical Package for Social Sciences (SPSS) Version 22.0 (IBM SPSS Statistics for Windows; IBM Corp., Armonk, NY, USA).

## Results

**Figure 1:**
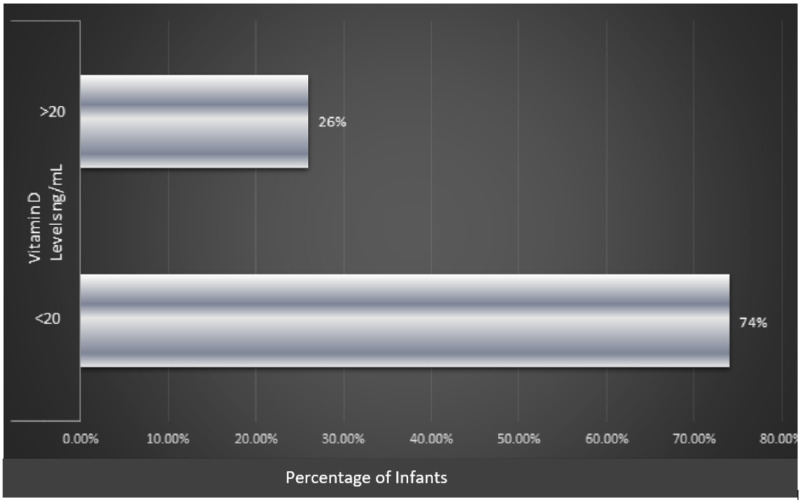
Prevalence of vitamin D deficiency among infants

A total of 200 infant-mother dyads were included in the study. There were more neonates than infants beyond the neonatal age in the study (80%, n=160 vs. 20%, n=40). The majority of infants were males (57.5%, n=115). Seventy four percent of the infants (n=148) had vitamin D levels below 20 ng/mL and 26% (n=52) had vitamin D levels over 20 ng/mL. Therefore, the prevalence of vitamin D deficiency among infants in our study was 74% (Figure 1). 

Among those infants with vitamin D deficiency, 41.5% had severe vitamin D deficiency (vitamin D levels <10 ng/mL). When subgroup analysis was done, the prevalence of vitamin D deficiency was 79% in the neonatal age group (127/160), which was higher than the prevalence in those beyond the neonatal age group (67.5%, 27/40). Moreover, mean vitamin D levels were significantly lower in neonates than in the post-neonatal age group (p-value <0.001). All pertinent biochemical parameters are shown in Table 1.

**Table 1:** Mean and median values of biochemical parameters SD, standard deviation; IQR, interquartile range; ALP, alkaline phosphatase

Variables	Mean ± SD	Median (IQR)
Infant Vitamin D levels (ng/mL)	16.81 ± 12.50	13 (8 - 21.75)
Calcium (mg/dL)	8.96 ± 1.65	9 (8 - 10)
Phosphorus (mg/dL)	11.98 ± 6.68	11 (6 - 16)
ALP (U/L)	222.48 ± 109.49	203.5 (153.5 - 273.5)
Mother Vitamin D levels (ng/mL)	12.47 ± 8.83	10 (7 - 15.75)

About 14% (n=28) of children including neonates had vitamin D level between 20-30 ng/mL and 12% (n=24) of them had level more than 30 ng/mL. Only one-third of infants (31%, n=138) reported adequate sunlight exposure (15 to 30 minutes per day), and, interestingly, 108 infants among these 138 (78.2%) had vitamin D deficiency. A major proportion of infants (85%) were breastfed. Infants beyond the neonatal age group were analyzed for clinical features of vitamin D deficiency, and half of them had frontal bossing followed by craniotabes seen in 30% of infants. Likewise, rachitic rosary was seen in 10% of the infants. Other associated infant comorbidities comprised pneumonia (10%) and developmental delay (5%).

About 10.5% (n=21) of the infants had a weight below the 3rd percentile. A sizable proportion of infants (25.8%) had their length below the 3rd percentile, although most infants (74.2%) had a length in the 3rd to 50th percentile. Seventy-five percent had an occipitofrontal circumference (OFC) in the 3rd to 50th percentile, and 23.7% had it over the 50th percentile.

Looking at associated mineral abnormalities, the majority of the infants (81.5%, n=163) had hyperphosphatemia (serum phosphate more than 4.5 mg/dL). Hypocalcemia (serum calcium less than 8.5 mg/dL) was found in 27% of infants. Alkaline phosphatase levels were normal (less than 400 U/L) in the majority of infants (95.5%), and only 4.5% (n=9) had alkaline phosphatase levels more than 400 U/L. However, among these nine infants, five (55.5%) had vitamin D deficiency. Most infants were born at term (89%, n=178). Additionally, most infants were born appropriate for gestational age (76.5%, n=153). When the geographical area was taken into consideration, nearly one-third of the study infants (36%, n=72) were from the hilly area. Ten infants in our study were on anti-epileptic drugs, and eight of them had vitamin D deficiency. Among the infants beyond the neonatal age group with vitamin D deficiency, 10% had pneumonia, and 5% had developmental delays.

In the maternal data, 85.5% (n=171) of the mothers had vitamin D deficiency, of whom 54.5% had severe vitamin D deficiency. Only 14.5% of the mothers had vitamin D levels over 20 ng/mL. Among the maternal comorbidities, pregnancy-induced hypertension was most frequent (7.5%, n=15). Adequate maternal sunlight exposure (15 to 30 minutes) was present in less than half (47.5%, n=95) of the study population. Among the 105 mothers without sunlight exposure, 69.5% (n=73) had vitamin D deficiency.

Lower gestational age, lower socioeconomic status, and inadequate sunlight exposure in infants had a significant influence on mean vitamin D levels (p-value=0.032). Birth weight also had a significant impact on vitamin D deficiency (80% of infants with weight <3rd percentile had vitamin D deficiency (p-value <0.001). Similarly, eight out of 10 infants with length <3rd percentile had vitamin D deficiency (p-value <0.001). Vitamin D deficiency was similar among males and female infants. Breastfeeding versus formula feeding and total intake did not significantly influence the prevalence of vitamin D deficiency. Logistic regression analysis was utilized to predict the determinants of vitamin D deficiency (Table 2). 

**Table 2:** Logistic regression analysis of determinants of vitamin D deficiency in infants

Variable	β coefficient	Adjusted Odds ratio	P-value
Age	2.3	10.1	0.001
Socio Economic status	0.62	1.9	0.036
Mother Vitamin D status	2.4	10.7	0.001

Most families belonged to the lower middle class (47.5%, n=95), followed by the lower upper class (33%, n=66).

**Figure 2:**
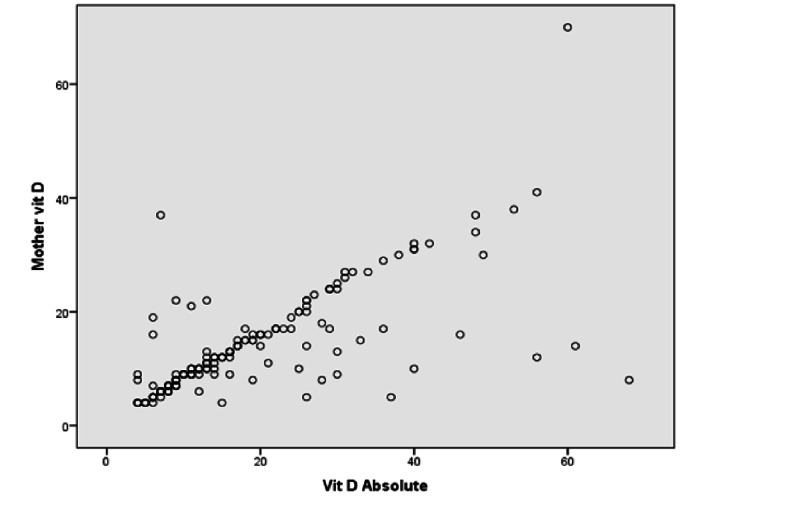
Correlation between maternal and infant vitamin D levels

Neonatal age, low socioeconomic status, and maternal vitamin D deficiency were independently associated with a higher risk of vitamin D deficiency in infants. A hundred and forty-four infants (84.2%) of 171 mothers with vitamin D deficiency also had vitamin D deficiency (p-value < 0.001). Likewise, there was a significant correlation (Pearson’s correlation coefficient of 0.724 and p-value <0.001) between the infant’s vitamin D levels and maternal vitamin D levels (Figure 2).

## Discussion

The current study reports the prevalence of vitamin D deficiency in infants from Uttarakhand, a northern state in India. In 2009, the IOF found hypovitaminosis D in 96% of neonates, 91% of healthy school girls, and 84% of pregnant women in North India [2]. In India, 50-90% of the population suffers from vitamin D deficiency due to inadequate exposure to sunlight and a lower dietary intake [4,5,7]. Our study reported a 74% prevalence of vitamin D deficiency among infants and 85.5% among mothers, which is comparable to previous studies. The US Endocrine Society has classified vitamin D deficiency as 25(OH) vitamin D levels less than 20 ng/mL, vitamin D insufficiency as levels 21-29 ng/mL, sufficiency as levels more than 30 ng/mL, and toxicity as vitamin D levels more than 150 ng/mL [3]. Similarly, the American Academy of Pediatrics (AAP, 2008) [8] and Institute of Medicine define vitamin D deficiency as serum 25(OH) vitamin D levels less than 15 ng/mL, mild to moderate deficiency as 5-15 ng/mL, severe deficiency as levels less than 5 ng/mL, and insufficiency as 16-20 ng/mL. They define sufficiency as levels between 21-100 ng/mL, excess as 101-149 ng/mL, and intoxication as levels more than 150 ng/mL [9]. The Kidney Disease Outcome Quality Initiative seconds the AAP in defining vitamin D deficiency as levels <15 ng/mL [10]. However, it defines insufficiency as levels between 16-30 ng/mL and sufficiency as levels of more than 30 ng/mL. None of our study infants had vitamin D levels >150 ng/mL. The current study used the US Endocrine Society definition for defining vitamin D deficiency.

Etiology

Vitamin D levels are maintained by dietary intake and by cutaneous synthesis [11,12]. It was shown that half an hour exposure to sunlight in naked infants and two hours exposure for fully clothed babies can sustain weekly calcidiol levels of 11 ng/mL and, thus, prevent severe vitamin D deficiency [13]. Even a brief exposure to direct sunlight for 10 to 15 minutes can produce 10,000 to 20,000 IU of vitamin D. Various factors influence vitamin D synthesis from sunlight, such as latitude, pigmentation, and area of skin exposed [2]. Hence infants and children with darker skin pigmentation are at risk for decreased vitamin D synthesis. These infants require five to 10 times longer duration of sunlight exposure for the synthesis of equal levels of 25(OH) vitamin D in comparison to children with lighter pigmentation [14]. To maintain sufficient vitamin D levels, Asian children require three times greater sunlight exposure than white Americans due to darker skin color. The AAP recommends avoiding direct sunlight exposure in infants less than six months of age [15], which can add to vitamin D deficiency [16-18]. However, neonates and infants have enhanced ability to produce vitamin D from sunlight when compared to adults due to higher surface area to volume ratio [19,20].

In our study, we have shown that the infants' sunlight exposure had a significant impact on vitamin D deficiency. Infants belonging to lower socioeconomic status had a higher prevalence of vitamin D deficiency than those from a higher socioeconomic status. This could be explained by the fact that children from lower socioeconomic status are likely to have reduced calcium and vitamin D intake, which was ascertained in our study [18]. Preterm neonates are at greater risk for vitamin D deficiency due to reduced placental transfer, inadequate exposure to sunlight, and decreased vitamin D stores due to low-fat mass. Of the 18 preterm neonates in this study, 15 had vitamin D deficiency.

Almost four-fifths of neonates and two-thirds of infants beyond the neonatal age group had vitamin D deficiency. There was a higher prevalence of vitamin D deficiency in neonates than in infants beyond the neonatal age group, which is similar to the IOF report [2]. The mean vitamin D levels were also significantly lower in neonates than in infants beyond the neonatal age group.

In our study, most infants with weight and height lower than the 3rd percentile had vitamin D deficiency. This can be well explained by lower nutrient intake, especially of calcium and vitamin D. Also, growth failure can be a manifestation of vitamin D deficiency [13]. Maternal vitamin D deficiency was shown to be an important risk factor for infants with vitamin D deficiency, which is similar to the findings reported by the IOF [2].

Predominant biochemical changes in infants with vitamin D deficiency included hyperphosphatemia, hypocalcemia, and hyperparathyroidism. This can be explained by immature renal tubular excretion in neonates, who were the predominant age group. Also, lower glomerular filtration rate, low intact parathyroid hormone (iPTH) levels, and renal tubular unresponsiveness to PTH, especially in the first three days of life, could have led to hyperphosphatemia alongside vitamin D deficiency. The coexisting metabolic bone disease, particularly among preterm small-for-gestational-age (SGA) neonates, could have contributed further to these biochemical changes. Alkaline phosphatase levels were normal (less than 400 U/L) in most of the infants from our study, and only 4.5% had elevated alkaline phosphatase levels of more than 400 U/L. This could be explained by the fact that during the neonatal period, the alkaline phosphatase levels do not change much [21,22].

Breast milk is a poor source of vitamin D (average 22 units/L; range 15-50 units/L) even in a vitamin D sufficient mother. Vitamin D supplementation of lactating mothers with 3000-6000 IU enhances the antirachitic effect of breast milk without leading to maternal hypervitaminosis D. Even in the current study, a major proportion of infants who were exclusively breastfed had a higher prevalence of vitamin D deficiency. After dietary intake, vitamin D is metabolized to calcidiol in the liver by the cytochrome P450 enzyme system. Medications such as carbamazepine, oxcarbazepine, phenobarbital, and phenytoin induce cytochrome P450 and enhance vitamin D metabolism and reduce the circulating active vitamin D concentrations [3,4,18]. These drugs can even cause hypocalcemia and precipitate seizures. Drugs such as corticosteroids and azole antifungals also affect the absorption, metabolism, or activation of vitamin D. As a very small proportion of the study infants and mothers were on anti-epileptic drugs, their influence on the prevalence of vitamin D deficiency could not be assessed.

Clinical features and morbidities

Vitamin D deficiency can go unnoticed without many clinical manifestations in the early phase and progress to florid rickets if left undiagnosed in older children. However, there may not be any clinical manifestations, especially skeletal changes in the neonatal age group, apart from the features of hypocalcemia [13]. In this study, 27% of the infants with vitamin D deficiency had hypocalcemia, and 10% had jitteriness. Vitamin D plays an important role in preventing respiratory infections [4], and 10% of the study infants beyond the neonatal age group had pneumonia along with vitamin D deficiency. Also, 5% had developmental delays. Frontal bossing, craniotabes, and rachitic rosary are important clinical manifestations of vitamin D deficiency in older infants and children [13], and these were seen in 50%, 30%, and 10% respectively, of the study infants who were beyond the neonatal age group. Our study ascertains exclusive breastfeeding and poor sunlight exposure as etiological factors for vitamin D deficiency. Likewise, the neonatal age group, low socioeconomic status, and maternal vitamin D deficiency were major independent determinants for Vitamin D deficiency in infants, as reported in previous studies [23,24].

Vitamin D deficiency is treated with vitamin D supplementation, either orally or intramuscular, along with adequate calcium supplementation to prevent hungry bone syndrome, which occurs due to underlying hypocalcemia and remineralization of the bone matrix. All the neonates and infants with vitamin D deficiency in our study were supplemented with calcium and vitamin D as per age-appropriate protocols. There are three oral forms of vitamin D, namely, ergocalciferol (25-hydroxyvitamin D2 or vitamin D2), cholecalciferol (25-hydroxyvitamin D3 or vitamin D3), and calcitriol (1,25(OH)2D). It is preferred to use vitamin D2 and D3 in infants and young children [18,25]. Calcitriol poses the risk of cholelithiasis and hypercalcemia and hence should be avoided as the first-line agent. It was reported that vitamin D3 has better efficacy in increasing serum 25(OH)D concentration (three times) than vitamin D2. Vitamin D2 or D3 is given at a dose of 1,000 IU daily in neonates, 1,000-5,000 IU daily for 12 weeks in infants, and up to 5000 IU daily in children older than one year, to achieve a total cumulative dose of 200,000 to 600,000 IU [4]. Children with malabsorption syndromes and those receiving anticonvulsants, glucocorticoids, antifungals, or antiretroviral medications need higher (1500 IU) and prolonged oral doses of vitamin D to achieve optimal serum levels [4,13,18].

Prevention

As found in this study, maternal vitamin D status has a significant impact on the infant’s vitamin D status. It is advised to assess 25(OH)D levels in all pregnant women and treat vitamin D deficiency with 3000-6000 IU of vitamin D3 until adequate serum levels of 25(OH)D (levels >20 ng/mL) are achieved [26-28]. Preterm infants require 400-800 IU of D3 per day from birth due to insufficient placental transfer of maternal vitamin D and other comorbidities associated with prematurity that may lead to a decreased intake or absorption of vitamin D [29]. In this study, all the mothers with vitamin D deficiency were also supplemented with vitamin D and calcium as per the unit protocol.

Strengths

Our study included a large sample size of mother-infant dyads. Extensive clinical assessment and biochemical workup was performed. This study aided in the early detection of infants with vitamin D deficiency and thus curtailed morbidity related to it. Detection and treatment of maternal vitamin D deficiency could prevent subsequent neonatal vitamin D deficiency.

Limitations

This is a single-center study and included only infants. The prevalence of vitamin D deficiency may vary depending on the region, and caution should be exercised in generalizing the study results.

## Conclusions

In our large group infant-mother dyads, the prevalence of vitamin D deficiency was significantly high. Neonatal age group, low socioeconomic status, and maternal vitamin D deficiency were independent determinants of vitamin D deficiency in infants. This high prevalence of vitamin D deficiency among infants in our region necessitates adequate nutritional support and supplementation for mothers and children to prevent morbidity associated with vitamin D deficiency.
